# Disseminated Histoplasmosis in Immunocompetent Individuals- not a so Rare Entity, in India

**DOI:** 10.4084/MJHID.2015.028

**Published:** 2015-04-20

**Authors:** Dibyendu De, Uttam Kumar Nath

**Affiliations:** Institute of Hematology and Transfusion Medicine (IHTM), Medical College, 88, College Street, Kolkata, India, PIN-700073

## Abstract

**Introduction:**

Histoplasmosis is a rare fungal disease caused by dimorphic fungi Histoplasma capsulatum. The causative fungus persists in soil, infects through inhalation and manifests in three main types-acute primary, chronic cavitary and progressive disseminated histoplasmosis. Disseminated Histoplasmosis (DH) is defined as a clinical condition where the fungus is present in more than one location. Among the forms of histoplasmosis, DH is the rarest and mostly found in an immuno-compromised individual.

Here we are presenting our experiences of the series of cases of DH in immuno-competent individuals who have been diagnosed in our institute in last 5 years.

**Materials and methods:**

This is a single centre retrospective observational study, conducted in Institute of Haematology and Transfusion Medicine, which is a referral centre for Eastern India, from May 2009 to April 2014. Only cases with DH in otherwise healthy immuno-competent individuals were included in the study. The histoplasmosis was confirmed either by presence of Histoplasma in biopsy specimen from an extrapulmonary organ or by positive growth in fungal culture

**Result:**

Total seven patients met the inclusion criteria. Five out of 7 patients were male. The mean age was 35 years. Five of the 7 patients presented with fever for a long duration. Six patients complained of significant weight loss before diagnosis. On examination, one patient had skin nodules, five patients had hepato-splenomegaly, and two patients had lymphadenopathy.

The laboratory investigations revealed anaemia in six out of 7 patients, and pancytopenia in 3 patients. Two patients had features of the hemophagocytic syndrome in the bone marrow.

All patients were treated with conventional amphotericin B deoxycholate and azole antifungal. One patient with adrenal involvement died in hospital. The patient with skin nodule had recurrent relapses. The other patients had resolution of symptoms and were clinically cured.

**Conclusion:**

DH is not an uncommon aetiology of fever of prolonged duration even in immuno-competent individual and should be kept as a differential diagnosis. Targeted investigation through early bone marrow biopsy and fungal culture may help in the diagnosis of DH. Imaging study to exclude adrenal involvement prevents case fatality. Cytopenia may be due to a secondary hemophagocytic syndrome, which improves with anti-fungal therapy. Treatment with either amphotericin B or itraconazole gives excellent outcome though therapy may have to be given for a prolonged period in case of relapses.

## Introduction

Histoplasmosis is a rare fungal disease caused by dimorphic fungi *Histoplasma capsulatum*. Two varieties of fungi are known to infect humans, namely: *H. capsulatum* var. *capsulatum and H. capsulatum* var. *duboisii.* Samuel Darling, first reported a case of histoplasmosis in an adult male presumed to have died of miliary tuberculosis.^1^

The organism exists in the mould (mycelial) form at soil temperatures and switches to the yeast form at normal human body temperatures (37°C). The causative fungus persists in soil, particularly in bird faeces. Birds do not transmit the disease; however, bird excretions contaminate the soil, thereby enriching the growth medium for the mycelium. Contaminated soil can be potentially infective for years.^2^

During outdoor activities, e.g. gardening, microconidia of the fungi are inhaled, they settle in the alveoli and are then ingested by the alveolar macrophages. The microconidia convert to the yeast form and replicate within the macrophages, and then spread to the regional lymph nodes, and throughout the reticuloendothelial system. The infected macrophages induce cytokines response and draw more macrophages and monocytes to fight the organism, and these coalesce together to form granuloma. The activation of the T-cell mediated immuno response is usually complete within two weeks and failure of this result in the progressive spread of infection to other organs.^3^

Clinical manifestations of histoplasmosis are of three main types-acute primary, chronic cavitary and progressive disseminated.^4^ Disseminated Histoplasmosis (DH) is defined as a clinical condition where the fungus is present in more than one location. Among the forms of histoplasmosis, DH is the rarest and mostly found in immuno-compromised individuals.

The disease is endemic in certain regions of Latin America and North America, and there have been cases reported in Europe and Asia. In India, Histoplasmosis is endemic in West Bengal and Assam, particularly in the Gangetic delta.^5^ Panja and Sen reported the first case of disseminated histoplasmosis from Calcutta in 1954.^6^ Later on many sporadic cases have been found both from North India as well as South India. This pattern of distribution may be related to climate, humidity level, and soil characteristics.

Here we are presenting our experiences of the series of cases of DH in immuno-competent individuals who have been diagnosed in our institute in last 5 years.

## Materials and methods

This is a single centre retrospective observational study. The study period is for 5 years, from May 2009 to April 2014. The data were analysed from the patient records of the adult patients aged more than 18 years, who were presented to Dept of Medicine or Dept of Haematology of Institute of Haematology and Transfusion Medicine, which is a referral centre for West Bengal as well as Eastern India. The catchment area of the institute includes Eastern and North-Eastern India as well as neighbouring Bangladesh, Nepal, Bhutan. Patients with acute pulmonary and chronic pulmonary histoplasmosis were excluded. Also patient with known immuno-compromised status like HIV infection, chronic liver or renal disease, chronic obstructive pulmonary disease (COPD), diabetes were excluded. Only cases with DH in otherwise healthy immuno-competent individuals were included in the study. The histoplasmosis was confirmed by either presence of Histoplasma in biopsy specimen from extra-pulmonary organ like bone marrow, liver, spleen, skin, adrenal gland etc or by positive growth in fungal culture from specimen from extra-pulmonary site obtained in sterile manner. Both bone marrow aspiration and biopsy were done in our institution and stained with Romanowsky stain, and Histoplasma was confirmed by the presence of PAS-positive intra and extracellular round bodies with refractile capsule surrounding it. Each aspiration and biopsy slide is reviewed by at least two hemato-pathologists independently.

## Result

Total seven patients met the inclusion criteria. Five out of 7 patients were male. The mean age was 35 years. Most of the patients were from the rural area. Only one patient was a farmer by occupation and had a definite history of exposure to soil.

Five of the 7 patients presented with fever for a long duration (Table 1). Six patients complained of significant weight loss before diagnosis. Three patients gave a history of cough, expectoration. One patient had a history of anorexia, vertigo and fall with severe weight loss. He had hypotension. Only one patient was pregnant while diagnosed to have DH. On examination, one patient had skin nodules involving the chest wall, which were multiple, 2–4cm size, coalescing with each other, and had yellow crust over it with ulceration. Five patients had hepato-splenomegaly, and two patients had lymphadenopathy.

The laboratory investigations revealed anaemia in six out of 7 patients, and pancytopenia in 3 patients (Table 2). All of them had a high ESR. The liver enzymes, (SGPT and SGOT), were raised in 5 out of 7 patients. No patients had increased bilirubin or abnormality in the renal function tests. Two patients had features of the hemophagocytic syndrome in the bone marrow.

**Figure 1:**
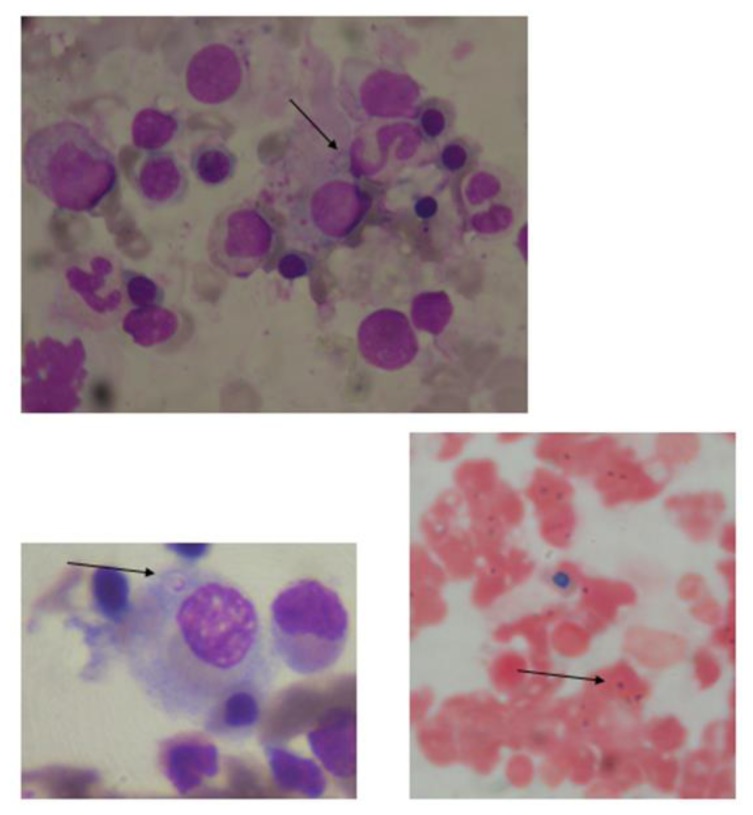
Intracellular histoplasma seen within macrophage, bone marrow aspiration smear, Romanowsky stain and PAS stain. oil immersion view, 100x.

In 5 of the 7 patients, the diagnosis of histoplasmosis was done by histopathological examination of bone marrow aspiration and biopsy (Figure 1). One patient, who had severe anorexia, weight loss, and hypotension, had bilateral adrenal mass. The biopsy, from adrenal mass, revealed Histoplasma on histopathological examination. Also, this patient had culture from bone marrow positive for Histoplasma. Though this patient did not have a hemophagocytic syndrome but presented with pancytopenia, possibly because of adrenal involvement. The patient with skin nodule and ulceration had both bone marrow histopathological diagnosis as well as culture from a swab of ulcer positive for Histoplasma.

None of the patients had any known condition for immuno-compromised status. The mean fasting blood sugar was 95 mg/dl. No patient had HIV, or any chronic liver disease, renal disease or COPD. No patient was on any chronic drug use. The mean CD 4 count was 708/cc.

**Figure 2:**
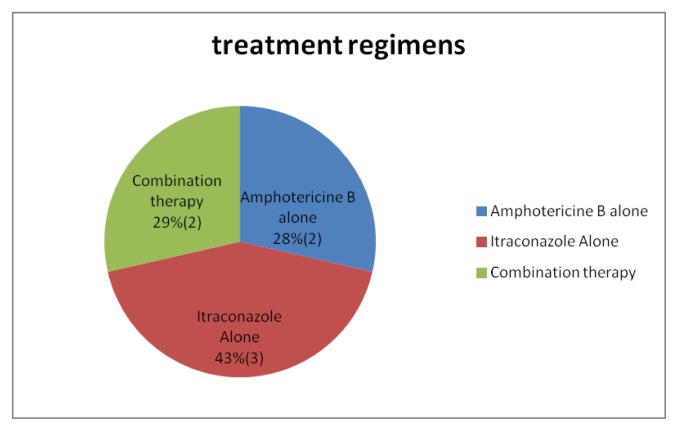
Treatment Regimens Used.

All of the patients had undergone treatment with either conventional amphotericin B deoxycholate or azole antifungal (itraconazole) or both. (Figure 2) Amphotericin B was given at a dose of 1–1.5 mg/kg/day for at least 4–6 weeks. None of the patients received liposomal Amphotericin B due to financial reason.

**Figure 3:**
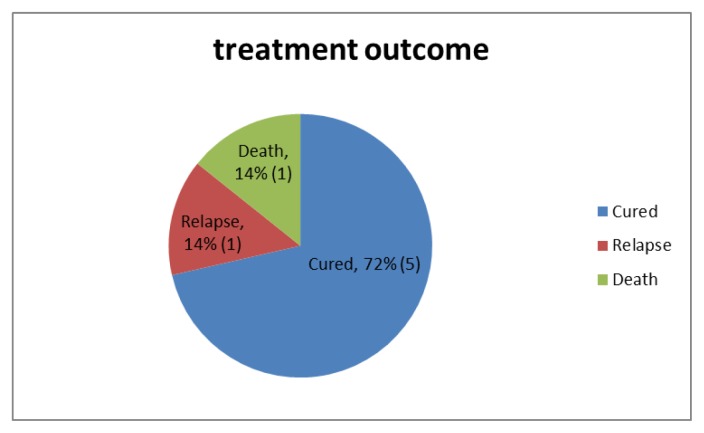
Treatment Outcome

One patient with adrenal involvement died in hospital. The patient with skin nodule had recurrent relapses even on itraconazole therapy, and her disease was controlled with a prolonged course of amphotericin B. The other patients had resolution of symptoms and clinically cured (figure 3). The patients with pancytopenia, two of them had hemophagocytic syndrome secondary to histoplasmosis, as evidenced by bone marrow aspiration. Their cytopenias also improved with therapy to Histoplasma.

## Discussions

Histoplasmosis is not an uncommon disease, in India, though is less commonly diagnosed and also under-reported. Three large studies, conducted previously from Delhi and South India, reported an occurrence of 37, 24 and 19 DH respectively, in 10 year follow up period.[7,8,9] The majority of patients in those studies were from Gangetic Delta, where it is considered endemic. The present study is the first largest case series from a tertiary care centre from Eastern India, which also points towards a higher prevalence. The high prevalence in the Gangetic Delta may be because of the specific soil type that is conducive for Histoplasma.

Disseminated histoplasmosis is known to occur in patients with immuno-compromised state. However, a previous study from CMC Vellore has shown a high prevalence of DH in immuno-competent patients too.^8^ Sometime histoplasmosis as a cause of prolonged fever remains undiagnosed. This study also highlights the incidence of DH as an important cause of pyrexia of unknown origin in immuno-competent individuals. Seventy-one percent of patients with DH presented with prolonged fever. Most of the patients are rural people (85%), and predominantly male, highlighting the importance of occupation and exposure to the soil as a clue to diagnosing DH.

Though previous literature describes a high prevalence of ulcerative lesions in about one-third of patients with DH, our study shows 14 % patient having ulcerated lesion. Symmers et al. in 1972 described a distinct clinical syndrome “Asian Histoplasmosis” with two salient features i.e. mucosal ulceration at mucocutaneous junctions or body orifices and a propensity to acute adrenocortical insufficiency.^10^ Our study did not show any such presentation.

Only one patient had adrenal gland involvement and died due to adrenal failure. Previous studies mention a high proportion (80%) of adrenal gland involvement detected via imaging studies and autopsy findings, but a clinical manifestation of adrenal insufficiency is uncommon, and occurs in only about 7–20%..^11^ Our study findings also corroborate with this report. So, though uncommon, imaging study and functional assay for latent or manifest adrenal failure need to be performed early in order to salvage the patient using early steroids concomitant with antifungal therapy.

Hematological abnormalities such as anemia, thrombocytopenia, and an elevated ESR are common and have been reported in the disseminated forms due to the involvement of the bone marrow. 12 In our study, 28% patients had a concomitant hemophagocytic syndrome, causing pancytopenia. The hemophagocytic syndromes improved with antifungal therapy. Previous reports described the presence of hemophagocytic syndrome associated with DH in immuno-compromised state especially in AIDS patients.^13^ However, our study confirmed the presence of secondary hemophagocytic syndrome even in immuno-competent individuals with DH.

Liposomal Amphotericin B is more effective and less toxic in the treatment of DH as compared to conventional Amphotericin B^14^ and produces more rapid clearance of fungemia. In our study, both Amphotericin-B Deoxycholate and Itraconazole were found to be effective in controlling the disease, with mortality in 1 patient only. However, 1 patient required prolonged therapy with Amphotericin B for recurrent relapses with Itraconazole.

## Conclusion

Disseminated Histoplasmosis is not an uncommon etiology of fever of prolonged duration even in immuno-competent individual in India, and should be kept as a differential diagnosis. Targeted investigation of early bone marrow biopsy and fungal culture may help in the diagnosis of DH. Imaging study to exclude adrenal involvement prevents case fatality. Cytopenia may be due to the secondary hemophagocytic syndrome, which improves with anti-fungal therapy. Treatment with either amphotericin B or itraconazole gives excellent outcome though therapy may have to be given for a prolonged period in case of relapses.

## Figures and Tables

**Table 1:** Clinical Features.

Clinical parameters		No. of patients (%) N= 7
Sex	Male	5 ( 71.4%)
	Female	2 ( 28.6%)
Mean age:		35 years
Residence	Rural	6 ( 85.7%)
	Urban	1 ( 14.3%)
Weight loss		6 ( 85.7%)
Fever		5 ( 71.4%)
Respiratory symptoms		3 ( 42.9 %)
Skin involvement		1 ( 14.3%)
Hepatosplenomegaly		5 ( 71.4%)
Lymphadenopathy		2 ( 28.6%)
Adrenal failure		1 ( 14.3%)

**Table 2:** Details of laboratory parameters of all patients.

Sl no.	Age (yr)	Sex	Hb (gm/dl)	TLC (/cmm)	Platelet count (/cmm)	ESR (mm/hr)	Hemo-phagocytic syndrome	Histoplasma in marrow	Treatment outcome
1	48	M	9.2	4500	170000	32	Nil	Y	Cured
2	31	M	8.4	1320	34000	62	Nil	Y	Died
3	40	M	8.2	3000	73000	48	Present	Y	Cured
4*	28	F	7.6	4850	210000	72	Nil	Y	Cured
5	30	F	12.2	4600	182000	28	Nil	N	Reccurent relapse
6	36	M	8.6	5800	178000	56	Nil	N	Cured
7	32	M	7.8	3100	67000	38	Present	Y	Cured

* This patient was pregnant.

Hb: Hemoglobin, TLC: total leucocyte count.
